# Validity and Reliability of the Spanish Version of Godin-Shephard Leisure-Time Physical Activity Questionnaire in Prostate Cancer Patients

**DOI:** 10.3390/healthcare13020154

**Published:** 2025-01-15

**Authors:** Javier Martín Núñez, Marie Carmen Valenza, Alejandro Heredia Ciuró, Andrés Calvache Mateo, Alba Navas Otero, Paula Blasco Valls, Gregory Reychler

**Affiliations:** 1 Department of Physical Therapy, Faculty of Health Sciences, University of Granada, 18016 Granada, Spain; cvalenza@ugr.es (M.C.V.); ahc@ugr.es (A.H.C.); andrescalvache@ugr.es (A.C.M.); albanavas@ugr.es (A.N.O.); 2 Oncological Radiotherapy Service of the “Hospital PTS”, Clínico San Cecilio University Hospital, 18016 Granada, Spain; javimartin29@correo.ugr.es; 3 Service de Pneumologie, Cliniques Universitaires Saint-Luc, 1200 Brussels, Belgium; gregory.reychler@saintluc.uclouvain.be; 4 Institut de Recherche Expérimentale et Clinique (IREC), Pôle de Pneumologie, ORL and Dermatologie, Université Catholique de Louvain, 1200 Brussels, Belgium; 5 Service de kinésithérapie, Cliniques Universitaires Saint-Luc, 1200 Brussels, Belgium

**Keywords:** validity, reliability, prostate cancer, physical activity levels, Godin–Shephard Leisure Time Physical Activity Questionnaire

## Abstract

Background: Prostate cancer is highly prevalent in Spanish men. Although physical activity has benefits on several factors in prostate cancer survivors, this is diminished after medical oncology treatment. Cancer-related fatigue is one of the main barriers to physical activity, leading to a decrease in activity levels in these patients. Therefore, it is essential to assess physical activity in an efficient and simple way in order to design rehabilitation programmes for this population. Objective: The aim was to translate and adapt the Godin–Shephard Leisure-Time Physical Activity Questionnaire (GSLTPAQ) for Spanish-speaking prostate cancer patients and to assess its validity and reliability using the International Physical Activity Questionnaire (IPAQ) as a reference standard. Methods: Following Beaton’s guidelines, the GSLTPAQ was back-translated and cross-culturally adapted. Validity and reliability were assessed with a sample of thirty prostate cancer patients. Internal consistency and test-retest variability were also evaluated. Results: The Spanish GSLTPAQ demonstrated excellent validity, with high correlations with the IPAQ for light (r = 0.924), moderate (r = 0.931), and vigorous activities (r = 0.882). Internal consistency was strong (Cronbach’s alpha: 0.845–0.950). Test-retest reliability showed strong intraclass correlation coefficients (ICCs) for all activity levels, indicating good reliability. Conclusions: The Spanish version of the GSLTPAQ is a valid and reliable tool for assessing physical activity in prostate cancer patients. It shows strong correlations with the IPAQ, excellent internal consistency, and test-retest reliability.

## 1. Introduction

Prostate cancer is one of the most common cancers in men worldwide, with a significant impact on morbidity and mortality. In Spain, prostate cancer is the most common cancer in men, with approximately 29,000 new cases diagnosed each year [1]. Regular physical activity has been shown to be an important protective factor, improving quality of life and reducing the risk of recurrence and mortality in prostate cancer survivors [2].

However, medical oncology treatments have a negative impact on the physical activity levels of the patients. During and after treatment, they experience a significant decrease in physical activity due to side effects such as fatigue, pain, and muscle weakness [3]. This reduction in physical activity not only negatively affects patients’ quality of life but may also contribute to increased morbidity and risk of cancer recurrence.

Therefore, physical activity should be a focus of rehabilitation for prostate cancer survivors. There is considerable evidence of the benefits of physical activity on strength, obesity, psychological distress, fatigue, and therefore quality of life [4,5]. Recent research on physical activity and prostate cancer survivors has paid increasing attention to the effects of physical activity on psychological and physical well-being, as well as the influence of fatigue on physical activity levels in this population [6,7].

It appears that the benefits of physical activity on quality of life are mediated by its influence on cancer-related fatigue, with an inverse proportional relationship between fatigue and quality of life [8]. Furthermore, the presence of cancer-related fatigue is one of the barriers to physical activity in prostate cancer survivors and therefore one of the main causes of reduced physical activity levels in this population [9]. Consequently, it is important to accurately assess physical activity levels in this population to design effective interventions to promote exercise and improve health outcomes [10].

Physical activity can be measured using different methods, including self-reported questionnaires and accelerometers [11]. Self-reported questionnaires are popular tools in epidemiologic research because of their low cost and ease of administration; among them, GSLTPAQ is one of the most important [12].

To address these challenges, it is critical to validate translated questionnaires against other validated instruments. The IPAQ is a widely used and validated standardized questionnaire for assessing physical activity in different contexts and populations [13,14]. However, this questionnaire has the difficulty of asking participants to estimate the duration of their physical activity in the last seven days, and it seems that cancer patients have some difficulty in remembering the exact time. On the other hand, the GSLTPAQ questionnaire only asks about times when physical activity lasted more than half an hour, so it is possible that it makes it easier for people with prostate cancer to remember [15,16].

Therefore, the aim of our study was to translate and cross-culturally adapt the GSLTPAQ questionnaire for Spanish prostate cancer patients and to assess its validity and test-retest reliability according to the Consensus-based Guidelines for the Selection of Health Measurement Instruments (COSMIN) using the IPAQ questionnaire as a reference [17].

## 2. Methods

We carried out the validation of this study in two parts. First, we performed a Spanish translation and cross-cultural adaptation of the GSLTPAQ, and second, we evaluated the validity and test-retest reliability of the GSLTPAQ in prostate cancer patients.

### 2.1. Questionnaire

The GSLTPAQ is a widely used instrument to assess the physical activity levels of individuals, particularly in the context of leisure-time activities. The GSLTPAQ measures the frequency of different intensities of physical activity, including mild, moderate, and strenuous activities. This questionnaire presented a moderate Pearson correlation coefficient between 0.53–0.57 in breast and leukemia cancer patients; however, there are no specific references in prostate cancer survivors [18,19]. By utilizing the questionnaire, researchers can effectively quantify and classify the leisure-time physical activity levels of individuals, providing valuable insights into their overall physical activity patterns and associated health outcomes [20].

Total physical activity levels are calculated using a specific formula: (frequency of light activity × 3) + (frequency of moderate activity × 5) + (frequency of strenuous activity × 9). Each type of activity is multiplied by a corresponding Metabolic Equivalent Task (MET) value to account for the different energy expenditures associated with each intensity level: mild activity has a MET value of 3, moderate activity has a MET value of 5, and strenuous activity has a MET value of 9. Individuals who report a moderate-to-vigorous leisure-time physical activity of 24 or more are classified as active, while those who report a moderate-to-vigorous leisure-time physical activity of 23 or less are classified as insufficiently active, which corresponds to an estimated energy expenditure of less than 14 Kcal/kg/week [15].

As a reference for the validation of the GSLTPAQ questionnaire, we used the IPAQ questionnaire previously validated in the prostate cancer population (which has a r = 0.98 and a sensitivity of 89%) [13]. The IPAQ is a standardized tool for measuring physical activity levels in populations. The questionnaire assesses the frequency and duration of various activities such as walking, moderate and vigorous physical activity, and sedentary behavior over the past week. To administer the IPAQ, participants recall their physical activity over the past seven days, detailing the time spent on various activities in domains such as work, transportation, household chores, and leisure. Results are interpreted using Metabolic Equivalent Task (MET) scores, where each type of activity is assigned a MET value: walking (3.3 METs), moderate activities (4.0 METs), and vigorous activities (8.0 METs). The total physical activity score is calculated by multiplying the minutes spent in each activity by the corresponding MET value, and then summing these values to obtain a MET minutes/week score. This score categorizes individuals into low, moderate, or high activity levels and helps assess compliance with physical activity guidelines [14,21].

### 2.2. Part 1: Translation and Cross-Cultural Adaption

Following Beaton’s guidelines, the GSLTPAQ was back-translated and cross-culturally adapted [22]. First, the original GSLTPAQ was independently translated from English to Spanish by two bilingual native Spanish speakers. One translator had a medical background, and the other was a professional translator with no medical background. In the next step, the two translations were compared and any discrepancies were resolved by consensus (synthesis). Finally, a native English speaker, independent of our study, translated the GSLTPAQ back into English. The committee of experts then checked the comprehensibility of this translation against the original version.

### 2.3. Part 2: Evaluation of Psychometric Properties of the GSLTPAQ

Before enrollment, patients were fully informed about the study’s objectives and procedures and provided their informed consent. The study received approval from a local research ethics committee (0084-N-20).

Prostate cancer patients were prospectively recruited from the Radiation Oncology Service of the “Complejo Hospitalario Granada”. Participants with prostate cancer volunteered for the study without receiving any financial compensation. They signed a written informed consent form in accordance with the Declaration of Helsinki and current Good Clinical Practice guidelines.

The inclusion criteria for participants were as follows: patients diagnosed with prostate cancer, fluent in Spanish, and over 18 years of age. Participants were excluded if they had mental, degenerative, or progressive disorders that would prevent them from participating in and understanding the tests. To ensure adherence to the inclusion and exclusion criteria, the study coordinator was present during the participants’ first visit.

### 2.4. Protocol

The GSLTPAQ and IPAQ questionnaires were completed by the subjects themselves following an explanation from the researchers. Initially, the questionnaires were completed in person to address any questions or doubts from the participants. After completing the questionnaires in person, patients were instructed to complete the same questionnaires on their own one week later, and we also considered the possibility of global change [23]. The patient global rating of change scale ensured that their health status had not changed significantly between the two measurements. Participants were provided with a telephone number to call if they encountered any difficulties completing the questionnaires.

### 2.5. Statical/Data Analysis

Statistical analyses were conducted using SPSS 28.0 for Mac (IBM Corp., Armonk, NY, USA). Descriptive statistics were utilized to summarize demographic parameters. The normality of data distribution was evaluated with the Kolmogorov–Smirnov test. Based on the distribution’s normality, data were presented as mean ± standard deviation or median and interquartile range. Appropriate parametric or non-parametric tests were applied for comparisons depending on the distribution of the data. According to Hulley et al. [24], we conducted an a priori power analysis that resulted in a total sample of 29 participants and a statistical power of 80% to detect a moderate correlation (r = 0.50). Furthermore, the previous study by Prince et al. [25] determined that the minimum sample to consider this moderate correlation between self-report and objective measures should be a minimum of 30.

The validity and reliability of the questionnaire were evaluated within the patient population with only one group. Construct validity was determined using correlation coefficient (Spearman (rho) or Pearson (r) depending on the distribution of the data) to assess the relationship between the GSLTPAQ weekday subtotal, weekend subtotal, and total score with the Sit-Q-7d.

Internal consistency was measured with Cronbach’s alpha coefficient, with a value of ≥0.70 indicating acceptable reliability. Test-retest reliability, reflecting the GSLTPAQ consistency over a 7-day period under stable clinical conditions, was evaluated using the intraclass correlation coefficient (ICC) [26]. A two-way mixed-effects model with absolute agreement for a single rater/measure ICC was employed. Reliability was interpreted according to Koo and Li’s criteria [27]: greater than 0.90 for excellent reliability, 0.75 to 0.90 for good reliability, and 0.50 to 0.75 for moderate reliability [28]. All ICC values are reported along with their absolute values and 95% confidence intervals. Bias in GSLTPAQ scores and the limits of agreement were assessed using the Bland–Altman method [29].

## 3. Results

We conducted a back-translation and cross-cultural adaptation of the GSLTPAQ. Based on the results obtained, the expert committee responsible for this procedure deemed the questionnaire to be comprehensible and acceptable for prostate cancer patients. There were no issues with understanding or non-responses from participants, and thus no questions needed to be omitted. A modification was made for better comprehension: the term “extenuante” was replaced with “fatigante”. The average response time for the test was low, always under 5 min, and it was consistently faster to complete than its comparator, the IPAQ questionnaire. The Spanish adaptation of the test is available in the Supplementary Material.

**Figure 1:**
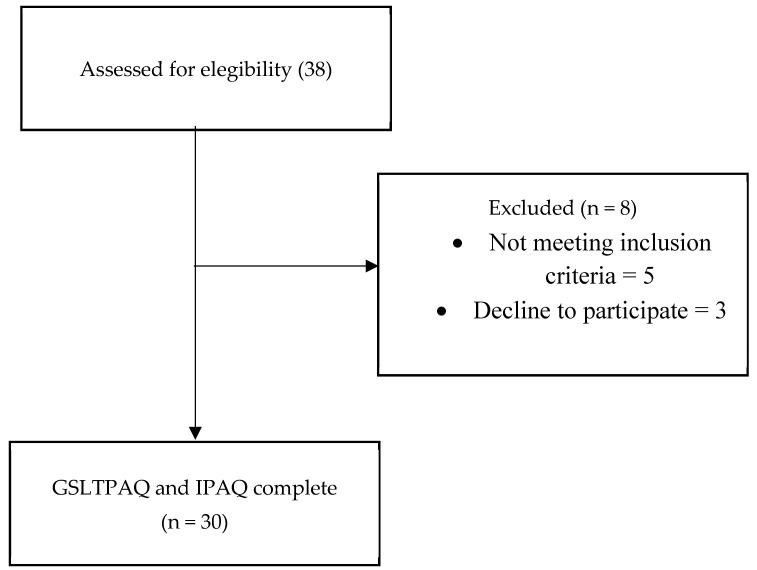
Flow chart of the study design. GSLTPAQ: Godin–Shephard Leisure-Time Physical Activity Questionnaire; IPAQ: International Physical Activity Questionnaire.

The flow diagram illustrates the inclusion of patients in our study (Figure 1), where a total of 38 prostate cancer patients were initially included, with 8 participants subsequently excluded (either due to refusal to participate (3) or not meeting the established inclusion criteria (5)). The descriptive data (Table 1) showed that the mean age of the participants was 69.47 ± 5.34 years, and the mean BMI was 28.01 ± 4.15. Regarding cancer status, the mean PSA level was 7.07 ± 6.28 and the Gleason score was 7.30 ± 1.08. In terms of cancer staging, the median score was 2 (0), and in most cases, there was no spread of cancer to nearby lymph nodes (N) or metastasis (M). Lastly, the Charlson Comorbidity Index had a median of 6.

Two measurements of the GSLTPAQ questionnaire were carried out (Table 2) with an intermediate week and the results of light, moderate, vigorous, and total physical activity were obtained, the result of the latter being 28 (0–76) kcal/kg/week in the first measurement and 30.50 (2–86) kcal/kg/week in the second measurement. The IPAQ questionnaire was also carried out in the first measurement, in which a total result of 3107.0 ± 671.4 MET min/week was observed.

### 3.1. Construct Validity

**Figure 2:**
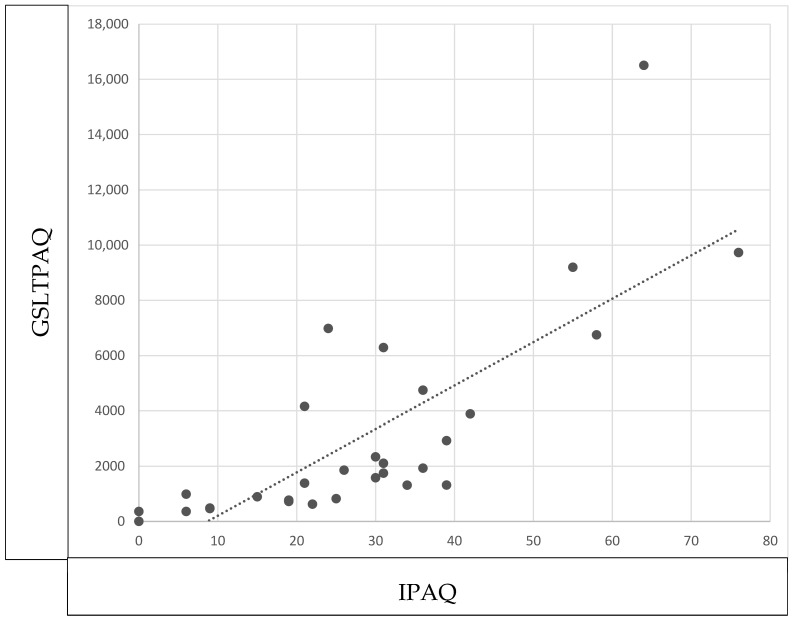
Construct validity of GSLTPAQ. Correlation between GSLTPAQ total score and IPAQ total score in prostate cancer patients.

Figure 2 showed an excellent correlation for light (r = 0.924; *p* < 0.001), moderate (r = 0.931; *p* < 0.001), and intense physical activity level (r = 0.882; *p* < 0.001); the same occurred with the total physical activity level (r = 0.815; *p* < 0.001).

### 3.2. Internal Consistency

The internal consistency was found to be excellent for the levels of light, moderate, and vigorous activity, as well as for the total results. The Chonbach’s alpha values obtained were 0.845 for light, 0.950 for moderate, 0.923 for vigorous, and 0.925 for total physical activity levels. In relation to the global change scale, there was practically no change between the two measurements with a median of 0.00 (0), and only in some patients was a brief improvement in the global state observed.

### 3.3. Test-Retest Reliability

**Figure 3:**
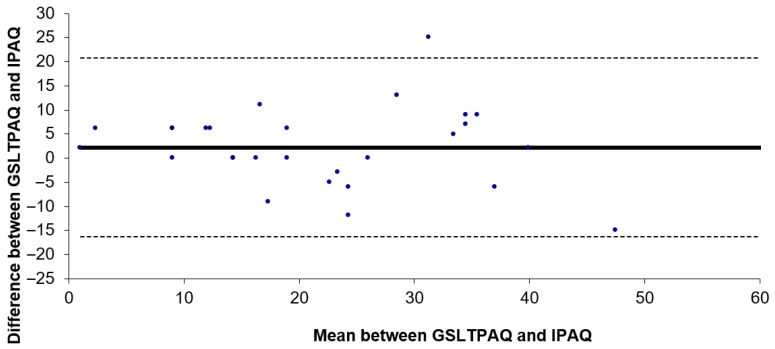
Bland–Antman plot of the GSLTPAQ (1) and GSLTPAQ (2) Spanish version of the Godin–Shephard Leisure Time Physical Activity Questionnaire.

A test-retest reliability study was conducted using the ICC for the subtotal scores of lights, moderate, and vigorous activity levels, as well as for total activity levels, with the GSLTPAQ over a 7-day interval. The results showed an ICC of 0.732 (95% CI: 0.509–0.863; *p* < 0.001) for light activity, 0.904 (95% CI: 0.809–0.953; *p* < 0.001) for moderate activity, 0.857 (95% CI: 0.721–0.929; *p* < 0.001) for vigorous activity, and 0.860 (95% CI: 0.728–0.931; *p* < 0.001) for total physical activity levels. The Bland–Altman plot (Figure 3) indicated that the bias between the two applications of the GSLTPAQ was 2.2. The limits of agreement for the difference between the total results of GSLTPAQ (1) and GSLTPAQ (2) ranged from −1.3 to 5.8 according to the Bland–Altman method.

## 4. Discussion

The aim of this study was to translate and culturally adapt the GSLTPAQ for Spanish-speaking prostate cancer patients and to evaluate its validity and test-retest reliability. The findings confirmed that the Spanish version of the GSLTPAQ for this patient group demonstrated statistically significant validity and test-retest reliability. The GSLTPAQ scores were correlated with those from the IPAQ questionnaire, validating the construct. Additionally, for prostate cancer patients, all subtotal scores and the overall GSLTPAQ score exhibited excellent internal consistency and test-retest reliability.

In our case, the GSLTPAQ questionnaire obtained a Pearson correlation coefficient for total activity of r = 0.815, and therefore a high correlation. However, our results show a lower correlation in prostate cancer survivors than in studies evaluating the questionnaire in breast cancer and leukemia. In these studies, a moderate correlation was observed (r ≤ 0.57) [18,19,30]. However, it should be noted that their comparison was with accelerometry and pedometer, unlike our comparison with another questionnaire.

We observed a high correlation in the GSLTPAQ questionnaire but, based on the review by Arimault et al. [11], it is possible that this is due to the overestimation of activity questionnaires compared to the use of accelerometers. Nevertheless, our results showed that prostate cancer survivors were not sufficiently active. However, this possible influence on the results obtained should be taken into account since we compared with another questionnaire instead of with accelerometry.

Our results showed that prostate cancer patients included in the study were classified as insufficiently active (≤23) based on moderate and vigorous activity according to the cutoff point. Our results are consistent with previous reviews that also show that prostate cancer patients do not reach these levels of physical activity [31]. Furthermore, although we measured activity levels by questionnaire rather than accelerometer, the previous review by Helmerhost et al. [32] demonstrated the feasibility of doing so.

This differs from previous studies that classified prostate cancer survivors as physically active. However, it should be noted that few studies have interpreted the results based on the score created by Godin et al. [15]. In many cases, researchers modify both the content (using the number of minutes of activity) and arbitrary cut-off points to create their own classification system [33,34]. Therefore, it would be of interest that future studies always use a standard classification system that allows the validity and reliability of this scale to be studied in other languages and populations.

Validity results of the Spanish GSLTPAQ questionnaire for prostate cancer patients showed to be consistent with previous studies conducted in other populations such as breast cancer and multiple sclerosis [11,35]. Based on our findings, we were able to demonstrate a strong correlation between the GSLTPAQ questionnaire and the IPAQ; therefore, we can determine that the GSLTPAQ questionnaire is a valid instrument to assess physical activity levels in prostate cancer patients. In line with our results, validations of GSLTPAQ in other languages such as Brazilian also showed significant results [36].

Our Bland–Altman analysis revealed a minor mean bias and narrow limits of agreement between the two GSLTPAQ applications, indicating strong concordance and minimal systematic variance. This approach effectively demonstrated the GSLTPAQ ’s reliability in consistently measuring activity levels. In line with our results, Ruiz-Casado et al. [13] conducted a validation and reliability study of the IPAQ and GPAC tests on Spanish cancer patients and observed consistent test-retest reliability.

## 5. Limitations

Limitations of our study include the fact that only a small geographical area of Spain was analyzed, and its external validity should be considered. Although the IPAQ comparator used is validated, it should be considered that accelerometry was not used as a comparator. It is possible that participants underestimated their level of physical activity; however, as this occurred in both tests, it does not seem to be reflected in the results obtained. The high BMI score should also be considered. It should be noted that the population included did not have comorbidities such as diabetes mellitus or hypertension; however, this high index should be considered when interpreting the results on activity levels.

## 6. Conclusions

We can therefore conclude that the Spanish version of the GSLTPAQ is validated and proves to be reliable in prostate cancer patients due to its excellent internal consistency, strong test-retest reliability, and good concordance, as reflected in the Bland–Altman analysis.

## Figures and Tables

**Table 1:** Characteristics of prostate cancer patients.

Characteristics	Cancer Patients (n = 30)
Age (y)	69.47 ± 5.34
BMI (kg/cm^2^)	28.01 ± 4.15
Charlson Index	6; 2–9
PSA	7.07 ± 6.28
Gleason Score	7.30 ± 1.08
Cancer Stage	2; 1–4
T	2; 0–4
N	0.0; 0–1
M	0.0; 0–1

**Table 2:** Results of GSLTPAQ and IPAQ questionnaires in prostate cancer patients.

Measures	Cancer Patients (n = 30)
GSLTPAQ light (1)	15 (0–21) kcal/kg/week
GSLTPAQ moderate (1)	2.50 (0–25) kcal/kg/week
GSLTPAQ vigorous (1)	0.00 (0–45) kcal/kg/week
GSLTPAQ total (1)	28 (0–76) kcal/kg/week
GSLTPAQ light (2)	15 (3–21) kcal/kg/week
GSLTPAQ moderate (2)	2.50 (0–35) kcal/kg/week
GSLTPAQ vigorous (2)	0.00 (0–36) kcal/kg/week
GSLTPAQ total (2)	30.50 (2–86) kcal/kg/week
IPAQ light	1170.4 ± 230.1 MET·min/week
IPAQ moderate	822.0 ± 407.2 MET·min/week
IPAQ vigorous	1130.6 ± 354.0 MET·min/week
IPAQ total	3107.0 ± 671.4 MET·min/week

GSLTPAQ: Godin–Shephard Leisure-Time Physical Activity Questionnaire; IPAQ: International Physical Activity Questionnaire; (1) First measurement; (2) Second measurement.

## Data Availability

Access to the database is available upon request by contacting the correspondence author.

## Supplementary Materials

The following supporting information can be downloaded at: https://www.mdpi.com/article/10.3390/healthcare13020154/s1, Figure S1: Spanish Adaptation of Godin Leisure-Time Exercise Questionnaire.
