# Supplementary material for: Validity and Reliability of the Spanish Version of Godin-Shephard Leisure-Time Physical Activity Questionnaire in Prostate Cancer Patients
Source: Healthcare (Basel). 2025 Jan 15;13(2):154. doi: 10.3390/healthcare13020154 (PMC11765051; doi:10.3390/healthcare13020154)
Supplement: Supplementary file 1 [file healthcare-13-00154-s001.zip › healthcare-3356460-supplementary.pdf]

Figure S1. Spanish adaptation of Godin Leisure-Time Exercise Questionnaire

GODIN LEISURE-TIME EXERCISE QUESTIONNAIRE

Durante un período típico de **7 días** (una semana), ¿cuántas veces de media hace los siguientes tipos de ejercicio **durante más de 15 minutos** en su tiempo libre (escriba en cada línea el número correspondiente)?

Puntuación semanal de actividad de ocio = (9 × Fatigante) + (5 × Moderada) + (3 × Ligera)

	Veces por semana		Total
**Ejercicio Fatigante**<br>(el corazón late rápidamente)<br>(por ejemplo: correr, trotar, hockey, fútbol, fútbol americano, squash,<br>baloncesto, esquí de fondo, judo, patinaje sobre ruedas, natación vigorosa, ciclismo vigoroso de larga distancia)		X9	
**Ejercicio Moderado**<br>(no agotador)<br>(por ejemplo: caminar rápido, béisbol, tenis, andar en bicicleta fácilmente,<br>voleibol, bádminton, natación fácil, esquí alpino, bailes populares y danza)		X5	
**Ejercicio Suave/Ligero**<br>(mínimo esfuerzo)<br>(por ejemplo: yoga, tiro con arco, pesca desde la orilla del río, bolos, herraduras, golf, motos de nieve, caminatas tranquilas)		X3	
Puntuación de actividades semanales en tiempo libre			

**Puntuación**	**Interpretación**
24 o más	Activo
14-23	Moderadamente activo
Menos de 14	Insuficientemente activo/sedentario
